# Distinguished Loop-Mediated Isothermal Amplification Assay to Detect Porcine Epidemic Diarrhea Virus Genotypes I and II

**DOI:** 10.3390/vetsci12050399

**Published:** 2025-04-23

**Authors:** Zhong Liu, Lanlan Li, Mengtao Fang, Xiaoqing Wei, Jieqiong Li, Qi Wu, Xiaoxue Yang, Yu Ye, Gen Wan, Dongyan Huang, Deping Song

**Affiliations:** 1 Department of Preventive Veterinary Medicine, College of Animal Science and Technology, Jiangxi Agricultural University, Nanchang 330045, China; 19970042875@163.com (Z.L.); 17371744121@163.com (L.L.); fmt15797711231@126.com (M.F.); 15579991756@163.com (X.W.); lijieq1125@163.com (J.L.); wuqi3950@163.com (Q.W.); yxx3336662001@163.com (X.Y.); yeyu@jxau.edu.cn (Y.Y.); 0000005341@jxau.edu.cn (G.W.); huangdongyan@jxau.edu.cn (D.H.); 2 Jiangxi Engineering Research Center for Animal Health Products, Jiangxi Agricultural University, Nanchang 330045, China

**Keywords:** porcine epidemic diarrhea virus, genotype, LAMP, detection

## Abstract

Porcine epidemic diarrhea virus (PEDV), a primary pathogen causing diarrhea in pigs, particularly in piglets, has a mortality rate of up to 100%. Field PEDV strains circulating in pig production can be phylogenetically divided into two genotypes, GI and GII. Differential diagnosis of clinical strains with different genotypes is helpful for understanding disease epidemiology, vaccine selection, and prevention and control measures. The loop-mediated isothermal amplification method (LAMP), a novel nucleic acid amplification technique, has been utilized to detect a variety of pathogens in practice. In this study, we developed a distinguished RT-LAMP method to identify genotypes GI and GII strains of PEDV. Two pairs of primers, PEDV-LM and PEDV-LS, were designed based on the membrane and spike genes of PEDV, respectively. PEDV-LM primers exhibited specificity for all PEDV strains, while PEDV-LS primers specifically targeted the PEDV genotype GI. The diagnostic sensitivity of both primers was 1 × 10^2^ copies/reaction, which is 100 times more sensitive than RT-PCR. The RT-LAMP reaction process was completed at 65 °C for 40 min just in a water bath or metal bath. A cross-reactivity assay confirmed that this method is specific for PEDV GI and GII, with no cross-amplification observed with other swine-origin viruses such as PDCoV, PoRV, PRV, and PRRSV. Therefore, this refined LAMP technique offers a rapid, sensitive, and reliable method with which to detect and differentiate between PEDV GI and GII, making it a superior tool for the large-scale clinical surveillance of PEDV infections.

## Simple Summary

Due to recombination or mutation among different porcine epidemic diarrhea virus (PEDV) strains, existing vaccines may exhibit reduced protective efficacy, resulting in significant economic losses. Consequently, developing a method to identify different genotypes of PEDV is of paramount importance. In this study, we established a loop-mediated isothermal amplification (LAMP) method for the differential diagnosis of PEDV GI and GII genotypes. Two sets of primers, PEDV-LM and PEDV-LS, were designed based on the M and S genes of PEDV, respectively. PEDV-LM exhibited specificity for all PEDV strains, while PEDV-LS specifically targeted the PEDV GI genotype. In the experiments, both sets of LAMP primers had detection limits of 1 × 10^2^ copies, which is 100 times more sensitive than RT-PCR. This assay demonstrated specific amplification of PEDV and PEDV GI genotypes without cross-amplification with other viruses. Additionally, the assay results can be visualized by adding nucleic acid dye. When tested on clinical samples, our novel method performed comparably to RT-qPCR. Therefore, this method can be used for the detection of different PEDV genotypes.

## 1. Introduction

Porcine epidemic diarrhea (PED) is a highly contagious devastating enteric disease in pigs, caused by porcine epidemic diarrhea virus (PEDV) [1]. PEDV was originally discovered in the United Kingdom in 1971, was first isolated in Belgium in 1978 [2], and is now prevalent worldwide [3,4]. In China, the first case of diarrhea caused by PEDV was initially documented in 1973, and the virus was discovered in 1984 [5]. In October 2010, a highly virulent variant strain of PEDV emerged in China, characterized by high morbidity rates (nearly 100%) and high mortality rates (80%~100% in infected piglets), resulting in enormous economic losses to the domestic pig industry in various cities and provinces [6]. In April 2013, the variant strain of PEDV emerged in the United States and caused the death of more than 8 million pigs within less than one year [7]. The main source of infection of PED is sick and carrier pigs, and the fecal–oral route is the core route of direct transmission [3]. Recently, according to the statistics of major animal diseases in China January 2025, the number of reported cases of PED ranked first among class II animal diseases (http://www.xmsyj.moa.gov.cn/yqfb/202502/t20250225_6470528.htm; accessed on 27 February 2025).

PEDV is an enveloped, single-stranded, positive-sense RNA virus that belongs to the genus *Alphacoronavirus* (α-CoV) in the family *Coronaviridae* of the order *Nidovirales* [8,9]. The genome of PEDV is approximately 28 kb in length, containing a 5′untranslated region (UTR), seven open reading frames (ORFs), and a 3′UTR with a polyadenylated tail, and is arranged in the order of 5′UTR, ORF1a/1b, spike glycoprotein (S), hypothetical protein gene (ORF3), envelope (E), membrane (M), nucleocapsid (N), 3′UTR [10]. Phylogenetic analyses based on the whole genome and S protein showed that all PEDV strains can be classified into two distinct genotypes: classical (GI) and variant (GII). The GI genotype is further subdivided into two subtypes, GIa and GI [11], and the GII genotype is further subdivided into three subtypes, GIIa, GIIb, and GIIc (or S-INDEL) [12]. At present, RT-PCR, RT-quantitative PCR (RT-qPCR), nucleotide sequencing, and phylogenetic analysis have been used to detect and differentiate GI and GII genotypes of PEDV. However, the application of these methods is limited somewhat by the expensive PCR equipment, long detection period, and high technical requirements, which makes it inconvenient in field detection and in differentiating the genotypes.

The loop-mediated isothermal nucleic acid amplification (LAMP) method with high DNA specificity was developed in 2000 as a novel nucleic acid amplification method [13]. This method’s use of the *Bst* DNA polymerase with high strand displacement activity leads to high sensitivity (less than 100 copies), and the reaction time is less than an hour under isothermal conditions [14]. Therefore, LAMP is simple and easy to implement once the appropriate primers are prepared and is less costly with just a water bath or metal bath. Thus far, the LAMP method has been used to detect and differentiate a variety of pathogenic etiologies, including the detection of CoVs [15,16,17] and the differentiation of PCV2a and PCV2b [18]. However, the LAMP method’s use to differentiate PEDV GI and GII genotypes has not yet been reported. In this study, a LAMP assay was developed to provide a rapid, sensitive, and reliable method for PEDV genotype detection.

## 2. Materials and Methods

### 2.1. Sample Collection

A total of 35 clinical samples (including feces and intestinal contents) were collected from Jiangxi Province, China, during the period 2022–2023 from suckling piglets 1–2 weeks old with severe diarrhea. Additionally, the viral strains utilized in this study, including PEDV (GI and GII strain), porcine deltacoronavirus (PDCoV), porcine rotavirus (PoRV), pseudorabies virus (PRV), and porcine reproductive and respiratory syndrome virus (PRRSV), were preserved in our labs.

### 2.2. DNA and RNA Extraction

Following the manufacturer’s instructions, genomic DNA from PRV was extracted using the TIANamp Genomic DNA Kit (TIANGEN, Beijing, China), while total RNA from the samples was isolated with RNAiso Plus (Takara, Dalian, China). The isolated RNAs and DNAs were dissolved in 30 μL of nuclease-free water. DNA samples were stored at −20 °C and RNA samples at −80 °C until use.

### 2.3. LAMP Primer Design

**Figure 1:**
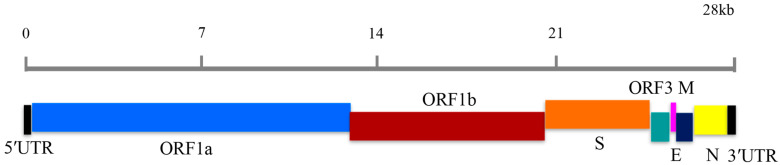
Schematic representation of genomic structure of PEDV.

The genome of PEDV contains seven open reading frames (ORF1a/1b, S, ORF3, E, M, N) (Figure 1) [10]. Utilizing the S and M gene sequences from the G1 and G2 genotypes of PEDV, multiple sequence alignments were performed. First, highly conserved regions in the M gene of the PEDV GI and GII genotypes were selected for the design of the PEDV-LM LAMP primer set, which is specific for all PEDV sequences. Subsequently, highly altered areas in the S gene of the PEDV GI and GII genotypes were chosen for the design of the PEDV-LS LAMP primer set, specific to the GI genotype sequence only. All the LAMP primers were designed with Primer3 v.0.4.0 (https://bioinfo.ut.ee/primer3-0.4.0/, accessed on 27 February 2025) and were synthesized with Sangon Biotech (Shanghai, China) with PAGE purification. These primer sequences are shown in Table 1.

### 2.4. Plasmid Standard Construction

The targeted RNA from PEDV was reverse-transcribed into single-stranded cDNA using random primers and M-MLV Reverse Transcriptase (RNase H-) (Takara, Dalian, China). Plasmids containing the target gene were constructed via PCR amplification using two sets of PEDV-LM F3/B3 and PEDV-LS F3/B3, respectively (Table 1). The amplified products were purified using the E.Z.N.A™ Gel Extraction Kit (Omega Bio-tek, Norcross, CA, USA) and subsequently cloned into a pMD19-T vector (Takara, Beijing, China). These plasmids were verified through sequencing, and the positive plasmids were extracted using the TIANprep Mini Plasmid Kit (TIANGEN, Beijing, China) according to the manufacturer’s instructions.

### 2.5. Conventional RT-PCR

The conventional RT-PCR method targeting the S gene was utilized to compare sensitivity. The reaction conditions were as follows: initial denaturation at 94 °C for 5 min, followed by 35 cycles of 94 °C for 10 s, 50 °C for 20 s, and 72 °C for 15 s, with a final extension at 72 °C for 5 min. The RT-PCR assay result was analyzed using 3% agarose gel electrophoresis, and then the product was subjected to purification, cloning, and sequencing procedures.

### 2.6. RT-LAMP

For both sets of primers used in the RT-LAMP assay, the final reaction volume was 25 µL. To optimize the reaction parameters, reactions containing different concentrations of dNTPs mix (0.6, 0.8, 1.0, 1.2, and 1.4 mM, Sangon Biotech, Shanghai, China), MgSO_4_ (2, 4, 6, 8, and 10 mM, New England Biolabs, Ipswich, MA, USA), each of the inner primers FIP and BIP (0.4, 0.8, 1.2, 1.6, and 2.0 µM), each of the outer primers F3 and B3 (0.2 µM), *Bst* 2.0 WarmStart^®^ DNA Polymerase (8 U, New England Biolabs, Ipswich, MA, USA), and 2.5 µL of template per reaction were tested. Additionally, the reaction systems were incubated at temperatures ranging from 59 °C to 69 °C for 30 to 80 min to determine the optimal temperature and detection time for the RT-LAMP reaction. The reactions were then terminated by heating at 80 °C for 5 min. After separation via electrophoresis, 1 µL of 4S Green Plus Nucleic Acid Stain (Sangon Biotech, Shanghai, China) was added to the RT-LAMP products, which were then analyzed electrophoretically on a 2.0% agarose gel. The detection results were also determined by the direct visualization of the color changes under UV light.

### 2.7. Sensitivity and Specificity

The specificity of the RT-LAMP assay for detecting PEDV GI and GII genotypes was evaluated using several significant pathogenic viral agents of pigs under the optimal condition, including PDCoV, PoRV, PRRSV, and PRV. Positive and negative controls were established with PEDV GI and GII genotypes and ddH_2_O, respectively. To determine the limit of detection (LOD) for the RT-LAMP assay, plasmid standards containing the S and M genes were used as templates. Additionally, the plasmid of the S gene served as the template for RT-PCR. All constructed plasmids in this study underwent serial dilution in tenfold increments, ranging from 1 × 10^8^ to 1 × 10^0^ copies.

### 2.8. Reproducibility

The reproducibility of the LAMP method was assessed using PEDV genotype-positive templates from GI, GIIa, and GIIb. Each template was tested three times under identical conditions. Electrophoresis was conducted to evaluate the amplified products on a 2.0% agarose gel.

### 2.9. Detection of PEDV in Clinical Samples by Conventional RT-PCR and RT-LAMP

A total of 35 clinical piglet samples, including one known positive sample each for PEDV GI and GII genotypes, were collected from Jiangxi Province. RNA was extracted from these samples and used as a template for RT-qPCR and RT-LAMP detection. Additionally, enzyme-free water was used as a negative control.

## 3. Results

### 3.1. Optimization of the PEDV RT-LAMP Method

**Figure 2:**
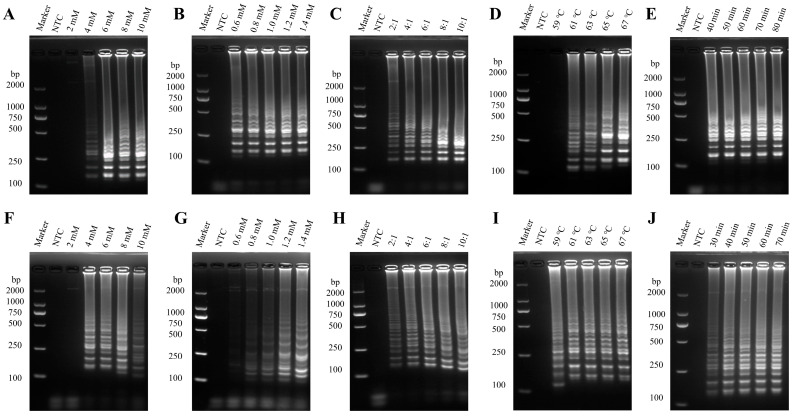
Optimization of RT-LAMP reaction conditions. (**A**–**E**) Optimizing the reaction conditions for the LAMP-LM system: from left to right, the order is the concentration of MgSO_4_ and dNTPs, internal-to-external primer concentration ratio, reaction temperature, and time. (**F**–**J**) Optimizing the reaction conditions for the LAMP-LS system: from left to right, the order is the concentration of MgSO_4_ and dNTPs, internal-to-external primer concentration ratio, reaction temperature, and time.

The optimized parameters for two RT-LAMP reaction systems were as follows: for the PEDV-LM system, the concentrations were 6 mM MgSO_4_ and 1.0 mM dNTPs, with 1.6 µM PEDV-LS-FIP and 1.6 µM PEDV-LS-BIP, and 0.2 µM of each outer primer F3 and B3. The reaction at 65 °C produced results in approximately 40 min (Figure 2A). In the second system, the concentrations were 8 mM MgSO_4_ and 1.4 mM dNTPs, with 1.6 µM of PEDV GI genotype-specific FIP and BIP primers. The reaction at 65 °C produced results in approximately 40 min (Figure 2B).

### 3.2. Specificity of the RT-LAMP Assay

**Figure 3:**
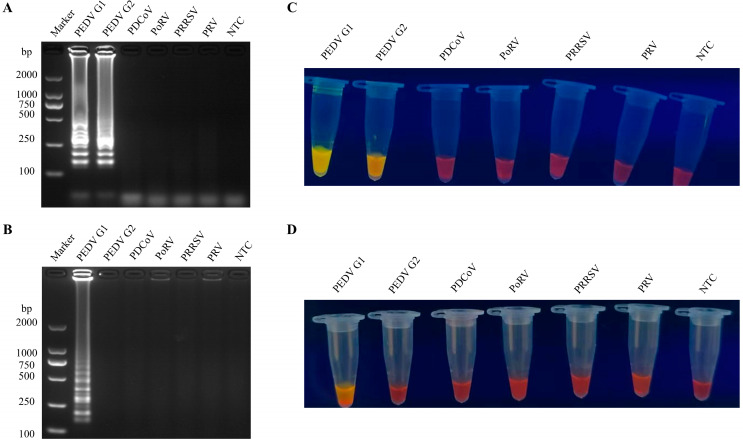
Evaluation of RT-LAMP specificity. (**A**) Specificity evaluation of the PEDV-LM system. (**B**) Specificity assessment of the PEDV-LS system. (**C**) Visualization results of the PEDV-LM system specificity evaluation. (**D**) Visualization results of the PEDV-LS system specificity evaluation.

In this experiment, the PEDV-LM exhibited ladder-like bands in the presence of PEDV-positive samples, whereas the PEDV-LS displayed these bands only in the presence of PEDV GI genotype-positive samples. Under identical conditions, neither set of LAMP primers produced a positive response from PDCoV, PoRV, PRRSV, or PRV (Figure 3A,B). The visualization detection results were consistent with those obtained from agarose gel electrophoresis (Figure 3C,D).

### 3.3. Sensitivity Comparison Between RT-LAMP and RT-PCR

**Figure 4:**
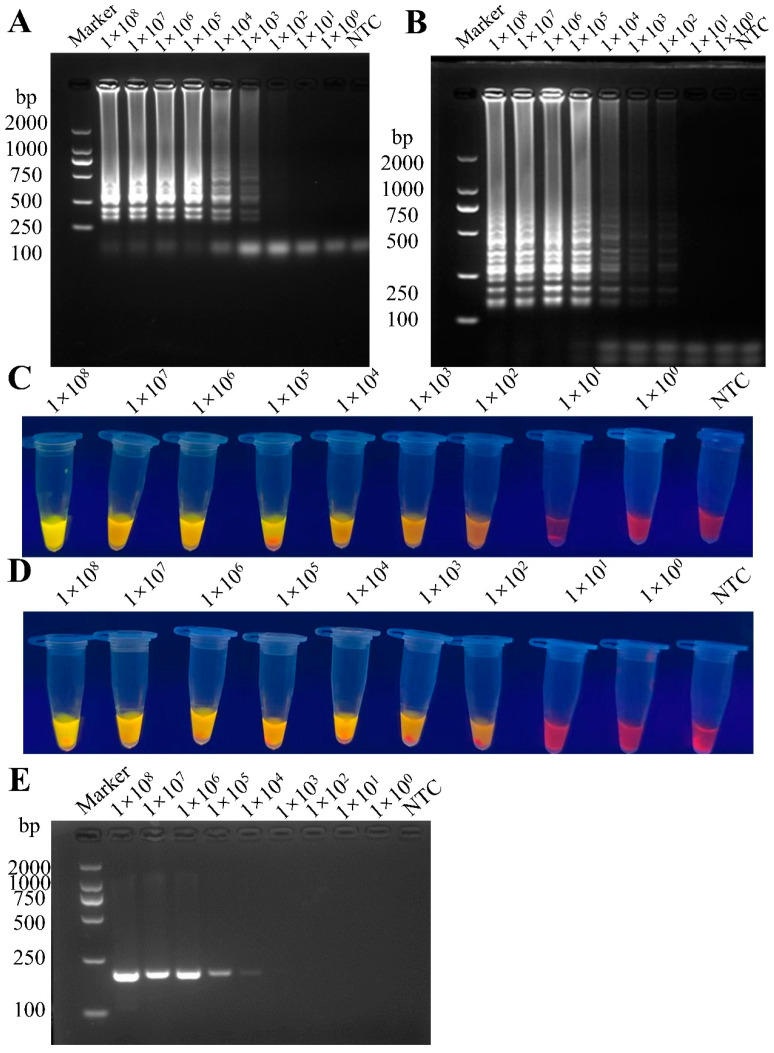
Comparison of the sensitivity of the RT-LAMP and conventional RT-PCR. (**A**) Sensitivity analysis of the PEDV-LM system. (**B**) Sensitivity analysis of the PEDV-LS system. (**C**) Visualization results of the PEDV-LM. (**D**) Visualization results of the PEDV-LS. (**E**) Sensitivity of conventional RT-PCR.

The LOD of PEDV GI and GII genotypes by LAMP were both 1 × 10^2^ copies/reaction from the results of visual observation and gel electrophoresis analysis (Figure 4A–D), while the LOD by RT-PCR was 1 × 10^4^ copies/reaction. (Figure 4E). These results indicate that the sensitivity of the LAMP method is 100 times higher than that of the RT-PCR method.

### 3.4. Reproducibility of the RT-LAMP Assay

**Figure 5:**
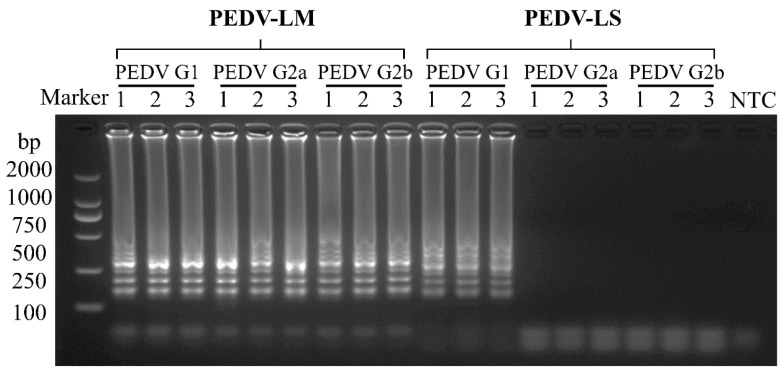
Reproducibility assay of the RT-LAMP by detecting various PEDV genotypes.

In this study, positive samples of PEDV genotypes GI, GIIa, and GIIb were utilized as templates for three replicates under each of the two sets of LAMP primers. Each positive template yielded identical results (Figure 5), demonstrating the stability of the LAMP method.

### 3.5. Clinical Sample Detection

**Figure 6:**
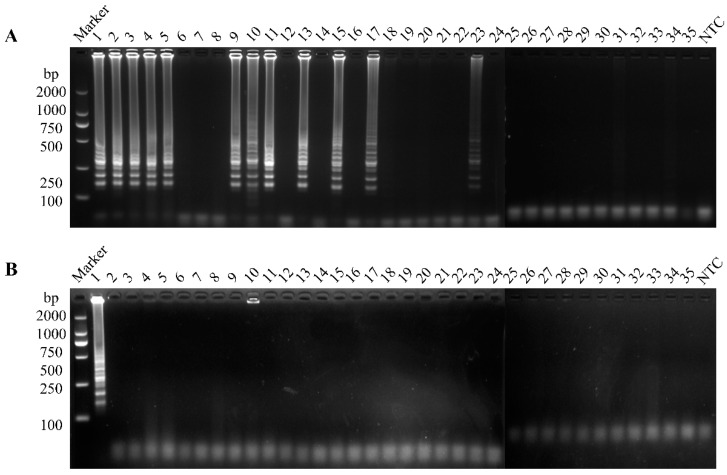
Detection of clinical samples by RT-LAMP. (**A**) The PEDV-LM system was used to detect 35 clinical samples. (**B**) The PEDV-LS system was used to detect 35 clinical samples.

To determine if their origins were contaminated with PEDV, 35 samples were examined using the RT-LAMP and RT-qPCR techniques. The results suggest that 12 positive samples were found by RT-LAMP (Figure 6A), of which 11 were PEDV G2 genotypes and 1 was a PEDV G1 genotype (Figure 6B), and this result was 100% consistent with RT-qPCR (Table 2).

## 4. Discussion

Since their first discovery in Europe in 1971 [6], PEDV infections have rapidly spread across Europe, Asia, and the Americas. The GII genotype of PEDV has dominated the circulating swine populations since 2010, causing significant economic losses to the swine industry [20]. Therefore, early diagnosis is crucial for controlling and preventing the spread of PEDV. Moreover, the rapid identification of the GI and GII subtypes of PEDV can facilitate the development of homologous vaccines for the prevention and control of these viral infections [21]. Thus, it is essential to develop a simple, rapid, and efficient method for distinguishing between the PEDV GI and GII genotypes.

Although qPCR diagnostic methods have been developed to differentiate between PEDV GI and GII subtypes [19], the application of qPCR requires expensive instrumentation and experienced technicians, which makes the application of this method relatively difficult in resource-poor regions. Loop-mediated isothermal amplification (LAMP) is an alternative technique that amplifies target DNA fragments at a constant temperature using 2–3 primer pairs, achieving results in a short time [13]. Moreover, LAMP primers are highly specific for target recognition [22]. Derived LAMP methods, such as AS-LAMP and PfSNP-LAMP, which utilize specially designed primers, can be employed for genotyping and SNP detection [23,24].

In this study, we developed a reverse-transcription loop-mediated isothermal amplification (RT-LAMP) method for the detection of porcine epidemic diarrhea virus (PEDV) genotypes GI and GII. Two sets of primers were designed to target the conserved region of the PEDV M gene and the hypervariable region of the S gene, referred to as PEDV-LM and PEDV-LS, respectively. The nucleotide homology of the M gene of PEDV has been shown to be 95.1–99.9%, indicating strong conservation within the PEDV genome [25]. In contrast, the S gene of PEDV exhibits high variability among different PEDV genotypes. Notably, the PEDV GI genotype has a 12-base deletion at nucleotide positions 167 and 175–185 compared to the GII genotype, which serves as a significant marker distinguishing these genotypes [26].

PEDV-LM was used for the detection of all PEDV strains, whereas PEDV-LS was employed to differentiate between PEDV GI and GII genotypes. Subsequently, we optimized the reaction conditions. The limit of detection (LOD) for both RT-LAMP assays was found to be 1 × 10^2^ copies, demonstrating a sensitivity that is 100 times greater than traditional RT-PCR. Furthermore, the RT-LAMP assay required only a constant temperature of 65 °C to facilitate the reaction, yielding results as quickly as 40 min.

Due to the specific design of LAMP primers targeting the M and S genes of porcine epidemic diarrhea virus (PEDV), no positive reactions were observed for other reference swine viruses, including porcine deltacoronavirus (PDCoV), Porcine Respiratory Virus (PoRV), porcine reproductive and respiratory syndrome virus (PRRSV), and porcine virus (PRV). This indicates that the method exhibits high specificity. Additionally, the LAMP assay was repeated three times using PEDV genotypes GI, GIIa, and GIIb as templates, yielding similar experimental results with their corresponding primers, thereby demonstrating the stability of the method. To assess the practical application of the LAMP assay, 35 samples from diarrheal piglets were tested. The results show that the positivity of RT-LAMP for detecting PEDV was consistent with qPCR. Furthermore, among the 12 PEDV-positive samples identified by RT-LAMP, 11 were positive for the PEDV GII genotype. These data also suggest that the PEDV GII genotype is the predominant genotype circulating in swine populations.

## 5. Conclusions

The LAMP detection method established in this study represents a valuable diagnostic tool. Compared to traditional PCR, it demonstrates higher sensitivity and does not require complex or expensive equipment. This makes it particularly well suited for diagnosing PEDV and differentiating its genotypes in resource-limited settings.

## Figures and Tables

**Table 1:** Primers for detection of PEDV GI and GII genotypes used in this study.

Primer Set	Primer ID	Sequence (5′-3′)
PEDV-LM	PEDV-LM-FIP	CCTGTACGCCAGTAGCAACCTTGAGCACCAACTGGTGTAACG
	PEDV-LM-BIP	ATTTCGTCACAGTCGCCAAGGCGACTGAACGACCAACACGT
	PEDV-LM-F3	TCTGTGATGGGCCGACAG
	PEDV-LM-B3	CCAGTGCCAGATGAAGCATT
PEDV-LS	PEDV-LS-FIP	ACTAGCAGTTTCAATGCCTGTGGTTACCTACCTAGTATGAACTCT
	PEDV-LS-BIP	GGCGTTCATGGTATTTTTCTCAGCCTAGGATCAAACGGCTCTTG
	PEDV-LS-F3	AAATTTAATGTTCAGGCACCT
	PEDV-LS-B3	GTGGCCTTATGTAAATAAAGCT
qPCR [19]	PEDV-GI-F	TGTTTTGGGTGGTTATCTACCTA
	PEDV-GI-R	AGCTGGTAACCACTAGGAT
	PEDV-GII-F	CCAGTACTTTCAACACTTAGCCTA
	PEDV-GII-R	GCCACTAGCAGTTGGATG

**Table 2:** Comparison of clinical sample detection results between RT-LAMP and qPCR methods.

PEDV Genotype	Sample No.	qPCR		RT-LAMP		Positive Rate	
		Positive	Negative	Positive	Negative	qPCR	RT-LAMP
GI	35	1	23	1	23	34.3%	34.3%
GII		11		11			

## Data Availability

Data will be provided upon request from the readers.
